# EAST MEETS WEST: MODELS OF "AGING IN COMMUNITIES": DEMENTIA, CAREGIVING, AND ACTIVE AGING BETWEEN THE US AND TAIWAN

**DOI:** 10.1093/geroni/igac059.1362

**Published:** 2022-12-20

**Authors:** TsuAnn Kuo, Fernando Torres-Gil

## Abstract

As countries around the world are entering the super aged societies, aging in place will need to be planned not only as an individual but also as a multi-disciplinary and age-friendly community. Many models are built based on the concept of "Aging in communities" in order to consider the multi-levels of needs and interests that older adults face when living in the same area. With Asia being the next fastest aging continent, innovative models and programs are also developed in Taiwan as it tries to promote healthy aging, combating dementia and supporting family caregivers. This symposium will include 4 papers by introducing a national program for frailty prevention, a community friendly model for people with dementia and a collaborative network to prevent family caregivers from being burned out. In addition, the village model developed in the U. S. will be compared to show how east meets west to demonstrate communities meeting the needs of older adults.

